# Risk factors of levodopa-induced dyskinesia in Parkinson’s disease: results from the PPMI cohort

**DOI:** 10.1038/s41531-018-0069-x

**Published:** 2018-11-16

**Authors:** Paolo Eusebi, Michele Romoli, Federico Paolini Paoletti, Nicola Tambasco, Paolo Calabresi, Lucilla Parnetti

**Affiliations:** 1 Neurology Clinic, Department of Medicine, University of Perugia, Ospedale S. Maria della Misericordia, Perugia, Italy; 2 IRCCS Santa Lucia, Rome, Italy

## Abstract

Levodopa-induced dyskinesias (LID) negatively impact on the quality of life of patients with Parkinson’s disease (PD). We assessed the risk factors for LID in a cohort of de-novo PD patients enrolled in the Parkinson’s Progression Markers Initiative (PPMI). This retrospective cohort study included all PD patients enrolled in the PPMI cohort. Main outcome was the incidence rate of dyskinesia, defined as the first time the patient reported a non-zero score in the item “Time spent with dyskinesia” of the MDS-UPDRS part IV. Predictive value for LID development was assessed for clinical and demographical features, dopamine transporter imaging (DaTscan) pattern, cerebrospinal fluid (CSF) biomarkers (Aβ42, total tau, phosphorylated tau, total α synuclein) and genetic risk score for PD. Overall, data from 423 PD patients were analyzed. The cumulative incidence rate of LID was 27.4% (95% CI = 23.2–32.0%), with a mean onset time of 5.81 years from PD diagnosis. Multivariate Cox regression analysis showed several factors predicting LID development, including female gender (HR = 1.61, 95% CI = 1.05–2.47), being not completely functional independent as measured by the modified Schwab & England ADL scale (HR = 1.81, 95% CI = 0.98–3.38), higher MDS-UPDRS part III score (HR = 1.03, 95% CI = 1.00–1.05), postural instability gait disturbances or intermediate phenotypes (HR = 1.95, 95% CI = 1.28–2.96), higher DaTscan caudate asymmetry index (HR = 1.02, 95% CI = 1.00–1.03), higher polygenic genetic risk score (HR = 1.39, 95% CI = 1.08–1.78), and an anxiety trait (HR = 1.02, 95% CI = 1.00–1.04). In PD patients, cumulative levodopa exposure, female gender, severity of motor and functional impairment, non-tremor dominant clinical phenotype, genetic risk score, anxiety, and marked caudate asymmetric pattern at DaTscan at baseline represent independent risk factors for developing LID.

## Levodopa: Predicting side-effect risk

Italian researchers identify seven independent risk factors for levodopa-induced dyskinesia in Parkinson’s disease (PD) patients. Long-term treatment with levodopa can cause uncontrolled, involuntary movements which adversely affect patients’ quality of life. Lucilla Parnetti and colleagues at the University of Perugia carried out a retrospective study of 423 patients enrolled in the Parkinson’s Progression Markers Initiative to determine factors that are predictive of levodopa-induced dyskinesia at the time of diagnosis. They found that the risk of developing dyskinesia was greater in patients who were female, carried PD-associated mutations, presented higher disease severity at the time of diagnosis or neuropsychiatric symptoms in early PD. Taking these factors into consideration in the very early phase of PD could not only improve management of the disease, but also the design of future biomarker studies and clinical trials.

## Introduction

Parkinson’s disease (PD) is a neurodegenerative disorder characterized by motor and non-motor symptoms.^1^ So far, levodopa still stands as the most effective symptomatic treatment for PD.^2^ However, long-term dopamine repletion treatment may lead to motor fluctuations, such as wearing-off and dyskinesias.^3,4^ Several factors participate in the development of motor fluctuations, including loss of dopaminergic neurons in the substantia nigra, changes in pre and post-synaptic striatal activity with chronic pulsatile stimulation of dopamine receptors,^5^ and the daily dosage of levodopa.^6^ Motor fluctuations highly impact on the quality of life of people with PD, representing a major criteria for eligibility to advanced treatments.^6^ Observational studies have shown that more than 50% of PD patients treated with levodopa for more than 5 years develop levodopa-induced dyskinesia (LID).^7^ Several risk factors for LID have been proposed, including levodopa dosage,^8^ treatment duration,^9^ female gender^10^ and low body weight.^11^ Other factors have been investigated as predisposing to LID, including neuroimaging findings, with conflicting results.^12–15^ It is worth noticing that the available studies are poorly comparable, given the different methodological approaches and follow-up duration, with patients being rarely followed up ever since de novo stage. The Parkinson’s Progression Markers Initiative (PPMI) is a large-scale international prospective observational study, started in 2010, designed to identify markers of disease progression in de novo PD patients. Clinical, neuroimaging and CSF/blood biomarkers are collected yearly. We wanted to define factors predictive of LID development already in de novo stage of PD.

## Results

### Cohort characteristics and LID incidence

**Fig. 1:**
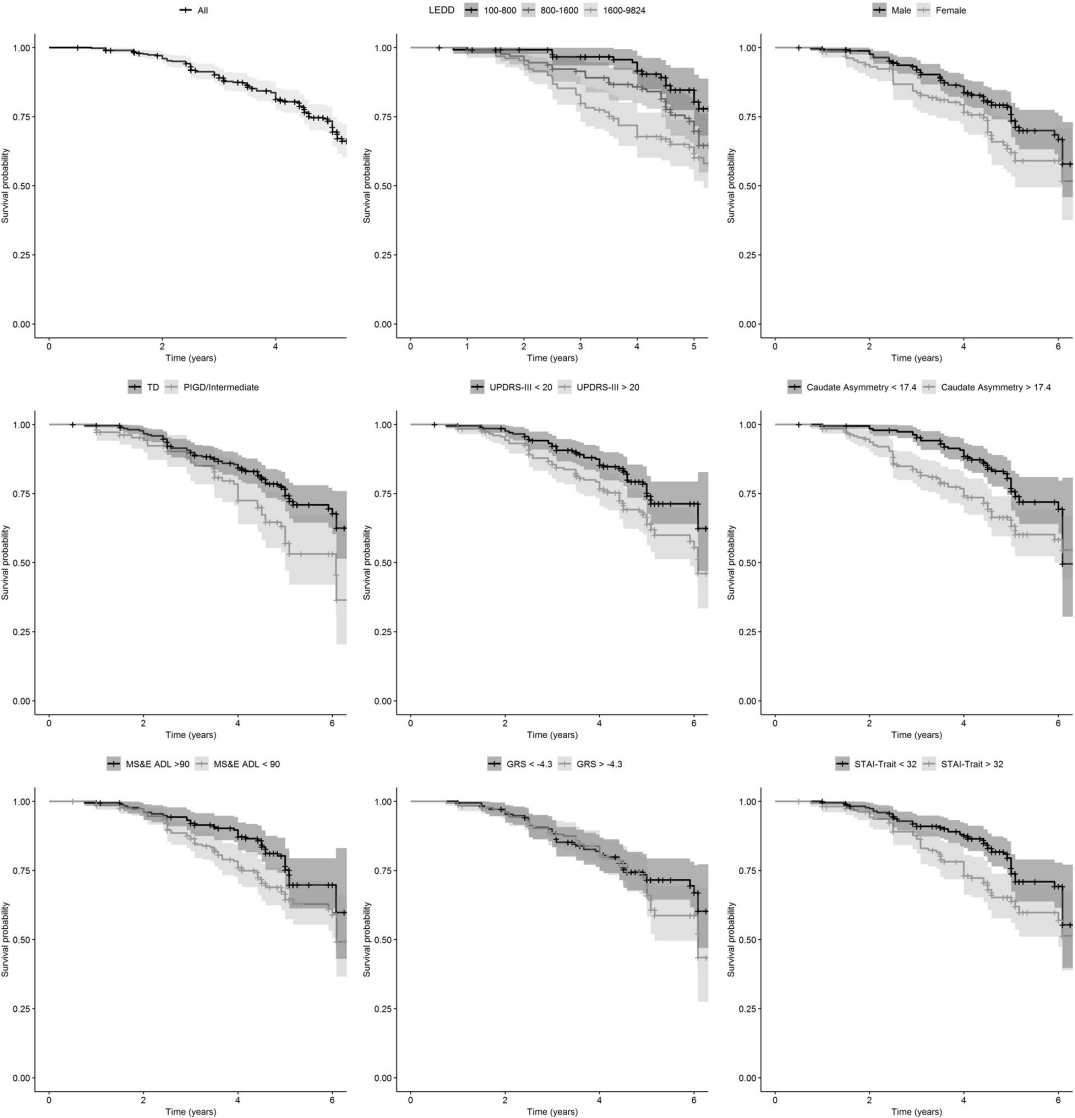
Kaplan–Meier estimates of survival without LID

Baseline demographic and disease characteristics of the cohort divided by the LID− (patients without LID) versus LID+ (patients developing LID) subgroups are presented in Table 1. At the time of data analysis, the median duration of follow-up was 4.6 years (min 0.0/max 6.4). Overall, 109/390 subjects experienced LID (27.9%, 95% CI 23.7% to 32.6%). In 33/109 patients experiencing LID, data regarding levodopa treatment and/or LID onset were missing. The median time to LID (Fig. 1) was 3.6 years (min 0.8/max 7.1) with an incidence rate of 64 per 1000 person-years.

**Table 1:** Baseline demographics, PD characteristics, DaTscan levels, and CSF analytes (β-amyloid 1-42 [Ab42], total tau protein [t-Tau], phosphorylated tau protein [p-Tau], and a-synuclein [α-syn]

Variable	LID− (censored events) *N* = 289	LID+ (observed events) *N* = 109	*p*-value
Age	62.32 ± 9.59	60.02 ± 10.00	0.040
Gender (female)	92 (31.8%)	45 (41.3%)	0.099
Education	15.43 ± 2.95	15.87 ± 2.85	0.170
Disease duration	0.58 ± 0.58	0.50 ± 0.46	0.161
Age at PD diagnosis	61.74 ± 9.56	59.52 ± 9.99	0.047
Family members with PD	73 (25.3%)	25 (22.9%)	0.714
MDS-UPDRS part III Score	20.25 ± 8.96	22.60 ± 8.34	0.015
Hoehn and Yahr			0.264
1	133 (46.0%)	41 (37.6%)	
2	155 (53.6%)	67 (61.5%)	
3–5	1 (0.4%)	1 (0.9%)	
PIGD Score	0.21 ± 0.21	0.27 ± 0.25	0.017
Tremor Score	0.49 ± 0.31	0.49 ± 0.34	0.964
PD clinical subtype			0.016
Indeterminate	20 (7.1%)	13 (12.1%)	
PIGD	47 (16.7%)	28 (26.2%)	
TD	215 (76.2%)	66 (61.7%)	
Side most affected			0.139
Left	114 (39.4%)	54 (49.5%)	
Right	169 (58.5%)	52 (47.7%)	
Symmetric	6 (2.1%)	3 (2.8%)	
Bradykinesia	238 (82.6%)	94 (87.0%)	0.365
Rigidity	213 (74.2%)	89 (82.4%)	0.115
Tremor	224 (77.5%)	84 (77.1%)	1.000
Modified Schwab and England ADL	93.53 ± 5.82	91.65 ± 5.89	0.005
UPSIT	21.96 ± 7.95	22.33 ± 9.00	0.708
SCOPA-AUT	15.13 ± 6.14	16.06 ± 6.46	0.197
Montreal Cognitive Assessment (MoCA)	27.07 ± 2.31	27.17 ± 2.41	0.695
REM Sleep Behavior Questionnaire	3.13 ± 2.11	3.41 ± 2.06	0.254
Epworth Sleepiness Scale	32.48 ± 10.22	5.87 ± 3.60	0.602
State-Trait Anxiety Inventory – State subscore	31.27 ± 8.98	34.48 ± 10.16	0.083
State-Trait Anxiety Inventory – Trait subscore	46.11 ± 4.05	34.71 ± 9.95	0.002
Geriatric Depression Scale (GDS-15)	2.13 ± 2.21	2.72 ± 2.78	0.046
Genetic risk score	−1.66 ± 0.86	−1.44 ± 0.97	0.052
Mean caudate uptake	1. 99 ± 0.55	1.95 ± 0.57	0.461
Contralateral caudate uptake	1.84 ± 0.54	1.76 ± 0.56	0.176
Caudate asymmetry index	17.71 ± 12.05	22.02 ± 14.15	0.005
Mean putamen uptake	0.83 ± 0.29	0.77 ± 0.26	0.052
Contralateral putamen uptake	0.69 ± 0.25	0.63 ± 0.21	0.001
Putamen asymmetry index	36.16 ± 24.47	39.79 ± 25.87	0.209
Aβ42	368.22 ± 101.55	370.55 ± 99.36	0.837
t-Tau	44.84 ± 18.63	43.19 ± 16.42	0.396
p-Tau	15.84 ± 10.74	14.96 ± 8.23	0.392
α-syn	1813.98 ± 772.17	1888.06 ± 790.48	0.406
LEDD	1347.51 ± 1255.83	2108.58 ± 1797.02	<0.001
Levodopa LEDD	713.73 ± 927.79	1491.64 ± 1595.91	<0.001
Dopamine Agonists LEDD	310.30 ± 491.45	263.42 ± 415.74	0.344
MAO-B LEDD	64.59 ± 71.59	66.68 ± 73.44	0.800
Amantadine LEDD	69.75 ± 160.50	120.64 ± 225.38	0.033

### Analysis of multiple risk factors for time to initiation of dopaminergic therapy

The average time for initiating a dopaminergic therapy was 1 year. Multivariate Cox regression analysis has shown that a combination of several factors was mildly accurate in predicting the initiation of dopaminergic therapy (Concordance = 0.61, 95% CI 0.57 to 0.64). The Cox proportionality assumption was validated with chi-square test for Schoenfeld residuals (overall *p*-value = 0.089); visual inspection of martingale residuals against individual covariates supported the linearity hypothesis. The final model included disease duration (HR = 0.81, 95% CI 0.66 to 1.00), Modified Schwab & England ADL score <90 (HR = 1.58, 95% CI 1.09 to 2.32), higher MDS-UPDRS part III (HR = 1.02, 95% CI 1.01 to 1.03), contralateral putamen (HR = 0.47, 95% CI 0.30 to 0.73), the polygenic risk score (HR = 1.18, 95% CI 1.04 to 1.34), and the anxiety trait as measured by STAI subscore (HR = 1.01, 95% CI 1.00 to 1.03).

### Analysis of risk factors for LID

The mean time for onset of dyskinesia after the initiation of any dopaminergic therapy was 3.6 years. The predictive value of each variable for investigating the incidence of LID was explored using Cox regression models, with total Levodopa equivalent daily dose (LEDD) included as covariate. Female gender was associated to a greater risk of developing dyskinesia (HR = 1.79, 95% CI 1.21 to 2.66, Table 2, Fig. 1). Several clinical characteristics were associated with incidence of dyskinesia such as Modified Schwab & England ADL (HR = 0.96, 95% CI 0.93 to 0.99, Table 2, Fig. 1), PIGD/Intermediate phenotype (HR = 1.75, 95% CI 1.18 to 2.59, Table 2, Fig. 1), MDS-UPDRS part III (HR = 1.03, 95% CI 1.01 to 1.05, Table 2, Fig. 1), and PIGD subtype (HR = 3.11, 95% CI 1.42 to 6.79, Table 2, Fig. 1). Interestingly, several DaTscan measures were associated with a greater risk of dyskinesia such as contralateral putamen (HR = 0.40, 95% CI 0.14 to 0.84, Table 2, Fig. 1), caudate asymmetry index (HR = 1.02, 95% CI 1.01 to 1.04, Table 2, Fig. 1), and putamen asymmetry index (HR = 1.01, 95% CI 1.00 to 1.02, Table 2, Fig. 1). In addition, onset of dyskinesia was associated with psychiatric features such as depression (HR = 1.11, 95% CI 1.04 to 1.19, Table 2) and anxiety as measured by STAI-State score (HR = 1.02, 95% CI 1.00 to 1.04, Table 2) and STAI-Trait score (HR = 1.04, 95% CI 1.02 to 1.06, Table 2, Fig. 1). None of the CSF biomarkers showed any predictive power.

**Table 2:** Cox regression analysis of individual risk factors adjusted for total LEDD

Variable	HR	HR 95% CI	*p*-value
Age	0.982	0.964–1.001	0.063
Gender (female)	1.794	1.212–2.656	0.004
Education	1.013	0.950–1.080	0.695
Disease duration	0.769	0.522–1.133	0.184
Age of PD diagnosis	0.983	0.965–1.002	0.073
Family history of PD	0.798	0.509–1.251	0.325
PIGD/Intermediate vs. TD	1.745	1.178–2.584	0.005
MDS-UPDRS part III Score	1.028	1.007–1.049	0.009
Hoehn and Yahr >1	1.409	0.952–2.085	0.087
Tremor Score	0.899	0.477–1.697	0.744
PIGD Score	3.109	1.424–6.790	0.004
Mean caudate	0.814	0.563–1.178	0.275
Contralateral caudate	0.724	0.496–1.057	0.094
Caudate asymmetry index	1.022	1.007–1.037	0.003
Mean putamen	0.529	0.246–1.138	0.103
Contralateral putamen	0.400	0.138–0.839	0.019
Putamen asymmetry index	1.008	1.000–1.016	0.043
Modified Schwab and England ADL	0.956	0.927–0.986	0.004
UPSIT	1.014	0.991–1.038	0.239
SCOPA-AUT	1.016	0.987–1.047	0.280
Montreal Cognitive Assessment (MoCA)	1.001	0.921–1.088	0.981
REM Sleep Behavior Questionnaire	1.045	0.953–1.146	0.351
Epworth Sleepiness Scale	1.005	0.950–1.063	0.862
State-Trait Anxiety Inventory – State subscore	1.018	1.001–1.036	0.042
State-Trait Anxiety Inventory – Trait subscore	1.037	1.018–1.056	<0.001
Geriatric Depression Scale (GDS-15)	1.111	1.037–1.191	0.003
Genetic risk score *100	1.200	0.961–1.469	0.107
Aβ42	1.001	0.999–1.002	0.601
t-Tau	0.999	0.987–1.010	0.802
p-Tau	0.994	0.974–1.015	0.579
α-syn	1.000	0.999–1.001	0.367
LEDD	1.233	1.120–1.357	<0.001
Levodopa LEDD	1.359	1.225–1.508	<0.001
Dopamine agonists LEDD	0.693	0.427–1.123	0.136
MAO-B LEDD	0.607	0.044–8.458	0.710
Amantadine LEDD	2.632	1.067–6.496	0.036

Hazard ratios with 95% confidence intervals and *p*-values

### Analysis of multiple risk factors for LID

Multivariate Cox regression analysis has shown that a combination of multiple factors was fairly accurate in predicting the onset of dyskinesia (Concordance = 0.74, 95% CI 0.681 to 0.80). The Cox proportionality assumption was validated with chi-square test for Schoenfeld residuals (overall *p*-value = 0.383), and visual inspection of martingale residuals against individual covariates supports the hypothesis of linearity. The final model included female gender (HR = 1.63, 95% CI 1.06 to 2.50, Table 3), 1000 mg/d of Levodopa LEDD (HR = 1.22, 95% CI 1.08 to 1.38, Table 3), STAI Trait score (HR = 1.03, 95% CI 1.00 to 1.05, Table 3), Modified Schwab & England ADL (HR = 0.97, 95% CI 0.93 to 1.01, Table 3), MDS-UPDRS part III (HR = 1.03, 95% CI 1.00 to 1.05, Table 3), PIGD/Intermediate phenotype (HR = 2.04, 95% CI 1.33 to 3.13, Table 3), caudate asymmetry index (HR = 1.02, 95% CI 1.00 to 1.03, Table 3), and the genetic risk score (HR = 1.35, 95% CI 1.05 to 1.74, Table 3).

**Table 3:** Cox regression analysis of the joint effect of multiple risk factors

Variable	HR	HR 95% CI	P-value
Gender (female)	1.61	1.05–2.47	0.030
1000 mg/d of Levodopa LEDD	1.26	1.12–1.42	<0.001
PIGD/Intermediate vs. TD	1.95	1.28–2.96	0.003
MDS-UPDRS part III	1.03	1.00–1.05	0.032
Modified Schwab and England ADL <90	1.81	0.98–3.38	0.061
Genetic risk score	1.39	1.08–1.78	0.010
Caudate asymmetry Index	1.02	1.00–1.03	0.037
State-Trait Anxiety Inventory – Trait subscore	1.02	1.00–1.04	0.012

Hazard ratios with 95% confidence intervals and *p*-values

### Random survival forests for LID

**Fig. 2:**
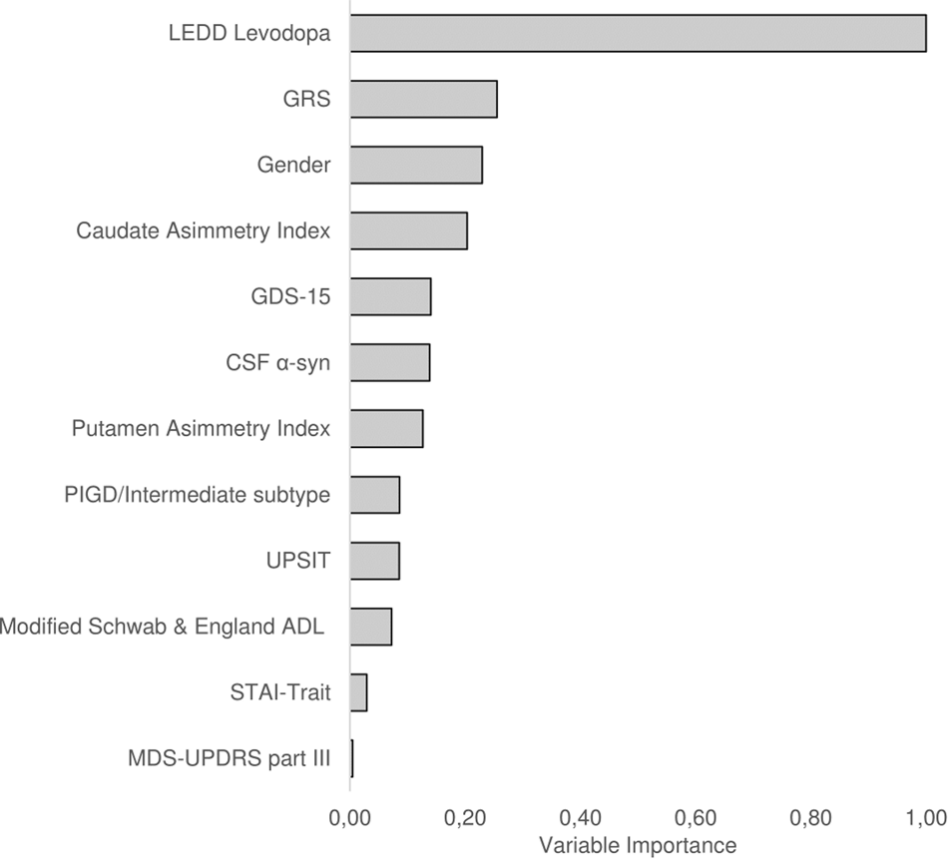
Ranking of variables according to relative importance in random survival forest model

After fitting random survival forests with 1000 trees and missing data imputation, 12 variables were selected using minimal depth criterion leading to an out-of-bag error of 24%. The final model was consistent with the results of multivariate Cox regression in terms of the selected variables. Using random survival forests, also CSF biomarkers entered in the panel of predictive factors (Table 2) for LID development, with α-syn ranking 6th according to the importance of all variables (Fig. 2).

## Discussion

LID negatively affects the quality of life of patients with PD. Despite extensive research, conflicting results have been gathered regarding modifiable and non-modifiable risk factors for LID development,^16^ with few studies assessing de novo patients. In this study, evaluating data of de novo PD patients included in the PPMI cohort, we identified a set of seven independent risk factors for LID development that can be taken into consideration already in the very early phase of the disease.

First of all, we confirm that female gender represents a crucial non-modifiable predictive factor for LID,^10^ independently from body weight and genetic factors.^10,11,17^ Second, our results underline that cumulative exposure to levodopa is positively associated with the development of LID, in line with the results deriving from other large cohorts of PD patients.^18,19^ Noteworthy, it is well known that levodopa accelerates the loss of nigrostriatal dopamine nerve terminals, a key pathophysiologic element in the development of dyskinesia. Dyskinesia in Parkinson’s disease are associated with changes in long term neuroadaptation and neuronal synaptic plasticity which in turn are linked to dopamine transporters and receptors density, respectively at a presynaptic and a postsynaptic level.^6^ Based on this concept, both positron emission tomography (PET) and single photon emission computed tomography (SPECT) were used to assess changes in neurotransmitter pathways involved in dyskinesia and, subsequently, to identify imaging biomarkers for LID development. Notably, lower dopamine transporter activity in the putamen evaluated by ^18^F-FP-CIT-PET in de-novo PD patients has been described as significant predictor of LID.^13^ Our results indicate that dopamine deficits in the contralateral putamen in de-novo PD patients is an independent predictor of a shorter time to levodopa initiation. Besides, both putamen asymmetry and caudate asymmetry indices evaluated by [^123^I]FP-CIT-SPECT significantly correlated with the development of dyskinesia, with the caudate asymmetry entering in the multivariate model. These findings reflect previous evidences indicating a positive relationship between the striatal asymmetric index and the magnitude of response to levodopa.^20^ Thus, PD patients with higher striatal asymmetric index show an increase of both response to levodopa and susceptibility to dyskinesia.

The probability of developing dyskinesia in Parkinson’s disease is also influenced by the initial clinical phenotype. Our findings showed that tremor-dominant (TD) phenotype is at lower risk of LID compared to PIGD or Intermediate. Previous studies have already demonstrated that tremor-dominant manifestation at disease onset is associated with a reduced risk of LID compared to rigid-akinetic (RA) phenotype.^21^ The reason of a lower risk of dyskinesia among TD patients may lay in different patterns of nigrostriatal denervation, morphologic lesions of the basal ganglia subregions and pathophysiological mechanisms between different phenotypes.^5^ Furthermore, TD patients usually show lower striatal dopamine depletion compared to RA patients on [^**123**^I]FP-CIT-SPECT,^22^ suggesting a less pronounced predisposition to LID. Nigrostriatal dopamine depletion is one of the main prerequisite for developing dyskinesia and dopaminergic denervation is increased by disease severity. Therefore, it is not surprising that disease severity at baseline represents an important predictive factor for LID. Our findings indicate that UPDRS Part III score in de-novo patients is a significant clinical biomarker to predict dyskinesia. However, it is not fully consistent with data from STRIDE-PD trial which showed UPDRS Part II score as risk factor for dyskinesia, whereas UPDRS Part III did not correlate with dyskinesia development.^19^ Such contrasting results may be due to the fact that UPDRS motor score does not always reflect precisely the status of presynaptic dopamine denervation evaluated by PET or SPECT.^23^ The relationship between the severity of disease and the susceptibility to dyskinesia is also supported by the significant inverse correlation we found between ADL Scale score and risk of LID. Thus, patients with higher disease severity testified by greater impairment on daily living activities are more prone to develop dyskinesia. Genetic susceptibility to dyskinesia is of great interest, with conflicting results deriving from several studies focusing on single-nucleotide polymorphisms (SNPs) of different genes. PPMI polygenic risk score was developed to explain the risk of idiopathic PD onset so far and it does not include several genes that are known to increase the risk of developing LID.^24^ However, to the best of our knowledge, for the first time in this study we highlighted a correlation between cumulative effect of known genetic risk variants of PD and LID development. Such results, if corroborated by extensive genome-wide association studies (GWAS), might represent a focus for future research. Interestingly, we also found that anxiety was associated to an increased risk of dyskinesia. This could be linked to the relationship between dopaminergic dysfunction and neuropsychiatric symptoms in early PD.^25^

Although CSF biomarkers did not explain LID onset in the Cox regression, we found that α-syn ranked 6th, according to the variable importance criterion, in the random survival forests model. This suggest that further investigations are warranted for exploring the role of CSF α-syn in predicting LID, with special attention to pay for the observation in larger cohorts or the measurement of oligomeric and phosphorylated forms.

There are limitations in our data, mainly due to the evaluation of dyskinesia. Even if specific scales have been validated to assess dyskinesia in PD,^26^ they were not used for evaluating qualitative and quantitative aspects of dyskinesia in the PPMI. Therefore, our results neither identified predictive factors for different types of dyskinetic complication in PD (chorea versus dystonia) nor found a possible explanation for the severity of dyskinesia.

In summary, our findings indicate that data deriving from a large cohort of de-novo PD patients monitored longitudinally are useful in understanding the composite aspects involved in the progression of disease. Our results highlight the role of several factors in determining dyskinesia, thus providing useful information for future design of both biomarker studies and randomized clinical trials.

## Methods

### Study design

Overall, 423 de novo PD participants were enrolled in the PPMI study between January 2011 and December 2012. Data were obtained from the PPMI database accessed 30 December 2017.

### Participants

The inclusion criteria for entering PPMI were: (i) age >30; (ii) presence of at least two parkinsonian signs such as bradykinesia, rigidity and resting tremor or have an asymmetric resting tremor, or asymmetric bradykinesia; (iii) having received the diagnosis not earlier than two years before enrollment; (iv) documented reduced striatal 123-I Ioflupane dopamine transporter (DatScan, GE Healthcare, Arlington Heights, IL) imaging binding consistent with PD; (v) no ongoing symptomatic therapy. Each PPMI participant received extensive assessment of motor and non-motor features.

### Standard protocol approvals, registrations, and consents

Each participating PPMI site received approval from an ethical standards committee on human experimentation before study initiation. Written informed consent for research was obtained from all individuals participating in the study.

### Baseline features

With the aim of defining predictive factors od LID development among *de novo* patients, we have investigated several variables available at baseline, including: (i) demographics (age, gender, family history, disease duration, education years); (ii) bradykinesia, rigidity and tremor were assessed according to UK Parkinson’s Disease Society Brain Bank Criteria (iii) motor features (International Parkinson’s disease and Movement Disorder Society-Unified Parkinson’s Disease Rating Scale (MDS-UPDRS)-Part II and III, total tremor score, postural instability–gait disturbance (PIGD) score, tremor/PIGD motor phenotype, Schwab-England activities of daily living (ADL) score, bradykinesia, tremor, and (iv) age/education adjusted Montreal Cognitive Assessment (MoCA); (v) non-motor manifestations (MDS-UPDRS-Part I), olfactory dysfunction via University of Pennsylvania Smell Identification Test (UPSIT) score, autonomic dysfunction via Scale for Outcomes in Parkinson’s disease-Autonomic (SCOPA-AUT) total score, REM sleep behaviour disorder (RBD) via RBD screening questionnaire (RBDSQ) score, sleep disturbances via Epworth Sleepiness Score (ESS), anxiety via State‐Trait Anxiety Inventory scores (STAI), depression via Geriatric Depression Scale (GDS-15); (vi) genetic risk score including 28 independent risk variants for PD that have been selected according to the results of a meta-analysis of PD genome-wide association studies,^24^ also including p.N370S in GBA and p.G2019S in LRRK2^27,28^; (vii) CSF biomarkers (CSF amyloid-β1-42, total (t)-tau, and phosphorylated tau (P-tau181) and a-synuclein); (viii) dopamine transporter imaging striatal-binding ratios (DATscan) (single-photon emission computed tomography (SPECT) with the DAT tracer 123I-ioflupane at baseline, with striatal-binding ratio calculated for left and right putamen separately using the occipital lobe as reference, obtaining ipsilateral, contralateral, mean measurements and asymmetry indices). The asymmetry index for caudate and putamen was calculated, according to PPMI indications for deriving variables, as the difference between left and right divided by the mean value.

### Levodopa exposure

Levodopa equivalent daily dose (LEDD) was reported for each participant reporting the initiation of dopaminergic therapy. We included total LEDD as the cumulative exposure to all dopaminergic drugs, as well as levodopa LEDD as the cumulative exposure to levodopa until the onset of dyskinesia (observed events) or the study exit (censored events).

### Levodopa-induced dyskinesia

The primary outcome was the incidence of LID, defined as the first time the patient reported a positive score in the item “Time spent with dyskinesia” of the MDS-UPDRS part IV.

### Statistical analysis

Statistical analyses were performed using R software version 3.4. Continuous variables were described by means and standard deviations, while categorical ones were reported as count and percentages. Predictors of LID onset were assessed using multivariate Cox proportional hazards regression models. In the absence of a conversion event, data were censored at the most recent clinic visit. We have first assessed the role of each risk factor in conjunction with the total LEDD. In order to avoid rejection of potentially important variables due to uncontrolled confounders, a p-value lower than 0.20 was used as screening criterion to consider the risk factor as candidate for the multivariate analysis. Backward elimination based on the Akaike’s information criterion was used to select a final model. Hazard proportionality was assessed through analysis of scaled Schoenfeld residuals whereas martingale residuals were plotted against continuous covariates to detect nonlinearity. As a complementary approach, we have applied random survival forests,^29^ a machine learning technique to determine important variables for the prediction of individual survival times. Variable selection was carried out using a conservative approach based on minimal depth criterion. Significance level of 5% was assumed for all the analyses.

## Data Availability

Data used in the preparation of this study were obtained from the PPMI database (www.ppmi-info.org/data). For up-to-date information on the study, visit http://www.ppmi-info.org.
